# Supplementary material for: The Social Stress Indicator (SSI)
Source: PLoS One. 2025 Sep 17;20(9):e0328768. doi: 10.1371/journal.pone.0328768 (PMC12443236; doi:10.1371/journal.pone.0328768)
Supplement: Attachment — Submitted filename: Response to reviewers_06_07_2025.docx [file pone.0328768.s001.docx]

Response to reviewers

| **Reviewer Comment** | **Feedback** |
| --- | --- |
| Introduction: Considering the relevance of the post-pandemic period in studying the impact of social media on mental health, would it not be appropriate to prioritise research published after this period? It is noted that more than 50% of the references are from before 2020. | That number reduced with the introduction of new references above 2024, and it must be noted that the earlier references are context specific to ideas in the field of which academic reference was given to the source of the ideas in those instances. |
| Introduction: It would be pertinent for the study to clarify the rationale for using synthetic data in your research. | This context was added in the methodology as synthetic data generated was specific to generic, rather than nuance content for the experimentation. As this study was conducted specific to SSI and its context to the indicator, future studies relate to the context specific research trained and used on real-world microblogs from a specific online discourse. |
| Introduction: Regarding the “state of the art”, which gaps does your research seek to address? | Added contextualisation and the relevant sources into what online social stress lacks, and what an SSI does differently to traditional approaches. |
| Materials and Methods: Lines 100-101: At present, there is a growing expectation that scientific knowledge should reach the general public and be accessible, thereby contributing to literacy and fostering an informed population. It would therefore be appropriate to explain the use of the three dimensions underpinning the Social Stress Indicator (SSI), namely sentiment analysis, subjectivity, and information-seeking behaviour. How were these dimensions/criteria identified or selected? | A detailed and referenced (using the latest relevant literature from 2024) was added to the section. |
| Ethics statement: Lines 113-114: Data were generated using a Large Language Model (LLM). What methodological precautions did you use to make these data reliable? | Context was added over the prompt contextualisation, as well as that every synthetic datapoint produced was vetted by the principal investigator. |
| SSS: Lines 135-136: "Additionally, five expert raters applied the rubric to synthetic microblogs generated to reflect diverse stress-inducing contexts". Could you present the eligibility criteria for the expert evaluators? It would also be appropriate to provide further details on the evaluation procedures followed by these experts. | **Context explained**. |
| Results: Lines 258-259: "SSI fails to capture high-stress events with sufficient sensitivity". Have you considered reflecting on the reasons behind the underestimation at high levels of stress, complementing your statistical analysis with qualitative approaches, such as the impact of cultural specificities? | Absolutely, and part of the SSI is the contextual relevance. I added a section explaining that the indicator would expressed elevated stress levels as compared to the Social stress score if weighting as well as topic contextualisation was included in the indicator. |
| Results: It would be appropriate to include a “Discussion” section in which the practical impact of the Social Stress Indicator is addressed — for example, its potential contributions to public policy, particularly in the field of mental health, in support of the 2030 Sustainable Development Goals, as well as the challenges associated with its implementation. | Results session is now “Results and Discussion”. A section was added on the practical impact of the SSI, but not based on the example provided. Given that the SSI would be best suited for social media, the practical implementation of the SSI for social media was recommended. |
| Conclusion: Specify the main lines of future research. For example, have you considered the possibility of cultural and linguistic adaptation of this indicator? | **Added in the recommendation**  “… linguistic nuances (like cultural and linguistic adaptation) …” |
| Minor Comments (Article structure): Line 103: Eliminate Social Stress Score, since in line 99, he has already presented, in full, the meaning of the acronym. On line 103, it must keep only "SSS". | **Changed.** |
| Minor Comments (Article structure): Line 109: An isolated "A" appears with no text. Is it a sentence that was cut and, by mistake, left the letter "A"? If yes, remove "A". | **Changed.** |
| Minor Comments (Article structure): Lines 128 and 141: Suggestion: Avoid that the chapter has the designation of Acronyms. For example, replace SSI and SSS with "Social Stress Indicator" and "Social Stress Score", respectively. | **Changed.** |
| Minor Comments (Article structure): Line 195: replace "for" with "For", as it is the beginning of a sentence. It must have been a mistake. | **Changed.** |
| Minor Comments (Article structure): Figures 1, 2 and 3 are barely noticeable and cannot be downloaded. Accessibility to them is suggested for a better analysis. | **Updated figures for quality. The download would be ready upon publication and not during the draft.** |
